# Surfactant proteins SP-B and SP-C and their precursors in bronchoalveolar lavages from children with acute and chronic inflammatory airway disease

**DOI:** 10.1186/1471-2466-8-6

**Published:** 2008-04-11

**Authors:** Oliver Tafel, Philipp Latzin, Karl Paul, Tobias Winter, Markus Woischnik, Matthias Griese

**Affiliations:** 1 Lung Research Group, Children's Hospital of Ludwig Maximilian University, Munich, Germany; 2 Division of Respiratory Medicine, Department of Paediatrics, Inselspital and University of Bern, Switzerland; 3 Praxis Karl Paul, Berlin, Germany

## Abstract

**Background:**

The surfactant proteins B (SP-B) and C (SP-C) are important for the stability and function of the alveolar surfactant film. Their involvement and down-regulation in inflammatory processes has recently been proposed, but their level during neutrophilic human airway diseases are not yet known.

**Methods:**

We used 1D-electrophoresis and Western blotting to determine the concentrations and molecular forms of SP-B and SP-C in bronchoalveolar lavage (BAL) fluid of children with different inflammatory airway diseases. 21 children with cystic fibrosis, 15 with chronic bronchitis and 14 with pneumonia were included and compared to 14 healthy control children.

**Results:**

SP-B was detected in BAL of all 64 patients, whereas SP-C was found in BAL of all but 3 children; those three BAL fluids had more than 80% neutrophils, and in two patients, who were re-lavaged later, SP-C was then present and the neutrophil count was lower. SP-B was mainly present as a dimer, SP-C as a monomer. For both qualitative and quantitative measures of SP-C and SP-B, no significant differences were observed between the four evaluated patient groups.

**Conclusion:**

Concentration or molecular form of SP-B and SP-C is not altered in BAL of children with different acute and chronic inflammatory lung diseases. We conclude that there is no down-regulation of SP-B and SP-C at the protein level in inflammatory processes of neutrophilic airway disease.

## Background

With each litre of air people inhale, the lung is exposed on average to at least one microorganism, in conjunction with particulates, antigens and possibly noxious gases [1]. In the lower lung, a thin film of surfactant in the epithelial lining fluid of the airspaces, represents the first line of defence to this burden of airborne pathogens and toxins. Pulmonary surfactant is a highly surface-active, complex mixture of approximately 90% lipid and 10% proteins [2,3]. Whereas the role of the surfactant proteins B (SP-B) and C (SP-C) as the critical surface tension lowering protein components of lung surfactant [4] are well established, their role in innate immunity is not yet.

SP-B is synthesized by alveolar type II cells to the mature 8 kDa protein and secreted into the alveolar space, where it is mainly found as a dimer [5]. Bronchoalveolar Clara cells also express pro-SP-B, but do not process it to the mature form. Instead, a 24 kDa proSP-B form is secreted into the alveolar space.

The main functions of SP-B are to increase the adsorption rate of phospholipids at the air-water interface and an involvement in the formation of tubular myelin together with SP-A and calcium [2,6]. Apart from that, anti-inflammatory properties as well as protection from oxygen-induced lung injuries have been described for SP-B [7,8]. SP-B has direct antibacterial activities; however this is inhibited by surfactant phospholipids and not selective to bacteria as also red blood cells are lysed, suggesting that endogenous SP-B may not play a significant role in alveolar host defense [9].

There are some data available on the levels of SP-B in the alveolar space, but almost no data on the molecular organization of SP-B and of pro-SP-B during various disease states [10]. Children and adults with bacterial pneumonia had unchanged levels of SP-B in their lavages [11,12]. Similarly, SP-B was unchanged in young and in adult patients with cystic fibrosis [13,14]. Infants with cystic fibrosis (CF) had the same SP-B concentrations in bronchoalveolar lavage fluid with or without active pulmonary infection or inflammation as controls [15]. SP-B content in lavage remained unchanged in Pneumocystis-pneumonia, but decreased significantly in ARDS and other forms of pneumonia [16]. SP-B was low in the BAL of patients at risk for ARDS before the onset of clinically defined lung injury, and in patients with established ARDS [17]. SP-B was lower in infants ventilated for severe RSV infection compared to healthy controls, ventilated post-surgery [18].

SP-C is exclusively synthesized by alveolar type II pneumocytes and secreted into the alveolar space [19]. It stabilises the alveolar surfactant film by enhancing the adsorption rate of phospholipids [20,21] and by increasing the resistance of surfactant against inhibition by serum proteins or oedema fluid [2]. In addition to these functions related to surface tension, SP-C may also be involved in host defense.

SP-C inserted into lipid vesicles interacts with a 12 residue non-transmembrane domaine with bacterial lipopolysaccharide; this enhances the association and the binding efficacy between lipopolysaccharide and CD14, a receptor on phagocytes [22,23].

Recently in various animal models of lung injury the regulation of SP-C expression by inflammation has been highlighted. SP-C concentrations were reduced in bleomycin induced lung fibrosis, hyperoxia, Aspergillus fumigatus and Pneumocystis jiroveci infections and a model of asthma, suggesting down-regulation of SP-C expression by pulmonary infection and inflammation. Unfortunately, there are not many ex vivo data available on the concentration and the molecular organization of SP-C in bronchoalveolar lavage of humans.

In adults, SP-C content in large aggregate surfactant remained unchanged in pneumocystis pneumonia, but decreased significantly in ARDS and other pneumonia [16]. Children and young adults with cystic fibrosis had increased SP-C [13,14]. Similarly, children with malignancies and immunosuppression during fever and pulmonary infiltrates, had two-fold increased levels of SP-C [24]. The available data contradict or only in part support the notion of a down-regulation of SP-C during pulmonary infection and inflammation.

The major goal of this study is therefore to investigate the concentrations and molecular forms of SP-B and SP-C and their pro-forms in the alveolar space under different infectious and inflammatory disease conditions.

## Methods

### Patients

Bronchoalveolar lavage fluid (BALF) from children and young adults with different chronic and acute inflammatory airway diseases including cystic fibrosis (CF), chronic bronchitis, acute pneumonia and a comparison group of subjects without lung disease (controls) was investigated.

CF was diagnosed by repeated sweat tests with elevated chloride concentrations together with the characteristic clinical presentation. 14 of 21 children were deltaF508 homozygous and 4 heterozygous. In 3 patients no mutation was detected by routine screen for the 30 most prevalent mutations in Germany. All patients were clinically stable, none had evidence for allergic bronchopulmonary aspergillosis or advanced hepatic disease. Some children with CF were part of the "bronchoalveolar lavage for the evaluation of anti-inflammatory treatment" (BEAT) study, a multicenter study to evaluate the effect of treatment with rhDNase on endobronchial inflammation, and were recruited in the Berlin and Munich centres [25]. Others underwent BAL for diagnostic purposes. Within the group of evaluated CF-patients no significant changes in the surfactant proteins and their quantitative appearance was observed between patients treated or not treated with rhDNase.

The 15 children in the chronic bronchitis group suffered from bronchitis without obstruction over a period of at least 3 months and endobronchial inflammation was diagnosed visually by bronchoscopy. Cystic fibrosis, primary ciliary dyskinesia, cellular or humoral immune deficiencies, gastro-oesophageal reflux disease and anatomic anomalies of the airways were excluded if clinically indicated. None of them had a pulmonary infiltrate in the x-ray or were diagnosed as having bronchial asthma (obstructive bronchitis).

Inclusion criteria for the 14 children in the pneumonia group were clinical symptoms (cough and/or fever) and a pulmonary infiltrate in x-ray and/or computer tomography. Exclusion criteria were interstitial pneumonia, immune deficiencies and malignancies as a reason for the pneumonia.

The 14 children of the control group did not have any illness with pulmonary affection. Here BAL was performed during anesthesia for elective surgery for minor conditions (excision of haemangioma, tonsillectomy, circumcision, hand surgery or groin hernia).

Clinical details of the children, the cellular results, total protein content and the microbiology results of the bronchoalveolar lavage are given in Table 1.

**Table 1:** Patients characteristic and bronchoalveolar lavage cells, protein content and bacteria

	Cystic fibrosis	Chronic bronchitis	Pneumonia	Healthy controls
n (male)	21 (10)	15 (5)	14 (9)	14 (9)
Age (years)	14.0 (11.7 – 17.3)	4.2 (0.8 – 7.2)	2.1 (0.9 – 4.9)	7.6 (2.2 – 23.3)
FEV1 (% pred)	89.0 (79.1 – 97.7)	100.0 (100.0 – 108.0)	96.0+	n.d.
Total cells (× 10^6^/ml)	7.3 (0.8 – 31.9)	14.5 (10.5 – 26.0)	20.5 (9.5 – 50.8)	9.2 (6.6 – 16.1)
PMN (%)	40.6 (2.2 – 79.8)	2.0 (1.0 – 7.0)	13.0 (2.5 – 56.0)	1.1 (0.8 – 2.0)
Total protein (μg/ml)	81.0 (51.4 – 130.2)	103.1 (85.8 – 166.1)	120.0 (88.6 – 159.5)	68.5 (51.7 – 87.9)
P. aeruginosa (x/n)	8/21	0/15, other 5/15	1/14, other 10/14	0/14
St. aureus (x/n)	7/21	0/15, other 5/15	1/14, other 10/14	0/14

Data are median and 25./75. percentile; n.d. not done; other (2 Pseudomonas, 2 Klebsiella, 1 Staphylococcus, 4 Pneumococcus, 6 Haemophilus, 1 E. coli (16 different bacteria, one patient had two different strains isolated); + Only one patient with pneumonia was assessed by lung function.

The study was approved by the ethics committee at the University of Munich. Written informed consent was obtained from the parents and/or the patients with appropriate age.

### Bronchoalveolar Lavage Procedure

BAL was performed using a 3.5 mm or 4.9 mm flexible bronchoscope in "wedge position" in a subsegment of the right middle lobe or the lingula. 4 × 1 ml/kg body weight saline (0.9%) at body temperature was instilled and immediately withdrawn by suction. The first aliquot of the recovered lavage fluid was treated separately. Fractions 2–4 were pooled and cleaned by gauze-filtration. The total cell count was measured by a haemocytometer and the differential cell count of the BALF by cytocentrifugation. The supernatant was used for the following analysis of total protein content and surfactant proteins.

### Surfactant Protein Analysis

**Figure 1:**
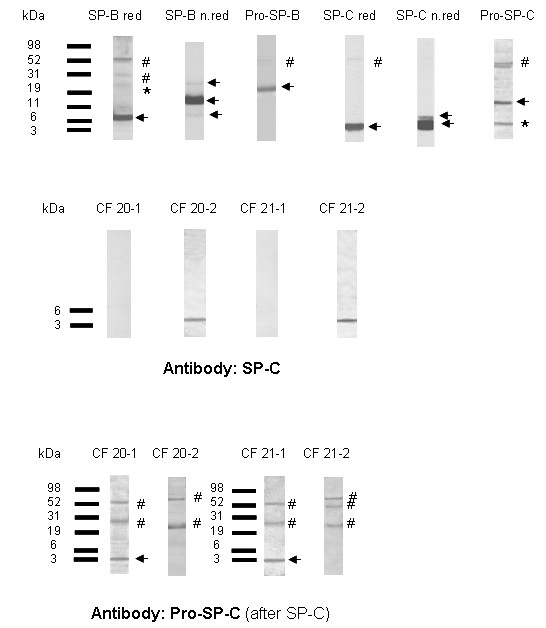
**(UPPER PANEL) Representative Western blots of SP-B, SP-C and their proforms.** Specific bands are marked with arrows. The frequency at which bands were detected at the respective molecular weights is listed in Table 2. Some blots also showed additional bands which were the residuals from previous incubations of the same blots with the other antibody (pro-SP-C was detected after the blot was incubated with SP-C, and SP-B after Pro-SP-B). In none of the patients pro-SP-C was detected. The arrow indicates the positive control for pro-SP-C in a lavage of a patient with a SP-C mutation and the presence of pro-SP-C in his lavage. **(MIDDLE PANEL) Initially in samples CF-20-1 and CF-21-1, no SP-C was detected.** Additional BALF samples from the same patients at later time points were available and run in addition. SP-C was only detected in samples CF 20-2 and CF 21-2. **(LOWER PANEL) Interestingly samples CF 20-1 and CF 21-1 showed a clear positive reaction with the pro-SP-C-antibody at a molecular weight of 3 – 4 kDa.** This might be fragments of pro-SP-C which reacted to the antibody or non-specific bands (arrow). All other bands in blot C) were non specific (#). * band from previous incubation # non-specific reaction.

Total protein content was determined according to the method of Bradford 1976 [26] with the BioRad Protein Assay Kid (BioRad, Richmond, CA, USA). For each patient four BALF samples containing 5 μg total protein each were prepared to detect (Pro-)SP-B and (Pro-)SP-C under reducing and SP-B and SP-C under non reducing conditions. The proteins were separated on NuPage 10% Bis-Tris gels using a Novex X-cell II Mini Cell system (Novex, San Diego, CA, USA) and then transferred onto nitrocellulose membranes by Western Blot in Nupage Blot modules (Novex, San Diego, CA, USA). Membranes with protein separated under reducing conditions were first incubated with pro-SP-B-antibody (recombinant anti-human from rabbit, C-terminal, charge 1/24/00, from Guttentag, USA) respectively SP-C-antibody (recombinant anti-human from rabbit, charge 22/96, from Byk-Gulden, Konstanz, Germany) and then with SP-B-antibody (recombinant anti-human from rabbit, charge C 329, from Byk-Gulden, Konstanz, Germany) respectively antibody against pro-SP-C (recombinant anti-human from rabbit, N-terminal, from Beers, USA). As second antibody we used peroxidase conjugated goat Ig-G anti rabbit from DIANOVA, Hamburg, Germany. The membranes were activated with enhanced chemiluminescence assay before exposing them to x-ray films (Hyperfilm ECL, Amersham Biosciences, Buckinghamshire, UK). After development the films were scanned with the FluorS Multi-Imager and the bands were analysed using the software-program "Quantity One". Apart from the qualitative evaluation a quantitative analysis was performed by multiplication of the optical density and the average diameter of each band. The comparison with standard curves (Figure 1) allowed approximate determinations of the amount of SP-B and SP-C in each patient sample.

### Statistical Analysis

Statistical analysis was performed with Graph Pad Prism Version 4.01 (San Diego, CA, USA) and Microsoft Excel 2000 (San Francisco, CA, USA), using Kruskal Wallis Anova for non-parametric results (given as median and 25. and 75. percentile) or Fisher's exact test for frequency distributions, corrected according to Bonferroni for multiple comparisons. Correlation coefficients were determined according to Spearman. A p-value of less than 0.05 was considered as being significant.

## Results

### Surfactant protein B (SP-B)

All 64 patients had SP-B in their lavages (Table 2). SP-B was uniformly present as a dimer, about half of the subjects also had monomers and few aggregates with the molecular weight of four SP-B molecules. Under reducing conditions, only monomers at about 8 kDa were present (Fig. 1A, Table 2). No differences between the four groups of subjects were found (Table 2).

**Table 2:** Frequency of detection of SP-B, SP-C and their proforms in bronchalveolar lavages at the indicated molecular weights determined by Western blotting

	SP-B			Pro-SP-B		SP-C		Pro-SP-C	
Western blot bands at (kDa)	8	16–18	30	19–21	24–25	4.2	7	3–4	15–16
CF (n = 21)	14 (67)	21 (100)	16 (76)	4 (19)	21 (100)	19 (90)	5 (26)	2 (10)	0 (0)
Chronic bronchitis (n = 15)	9 (60)	15 (100)	9 (60)	2 (13)	14 (93)	15 (100)	4 (26)	0 (0)	0 (0)
Pneumonia (n = 14)	4 (29)	14 (100)	6 (43)	1 (7)	12 (86)	13 (100)	2 (14)	0 (0)	0 (0)
Healthy controls (n = 14 for SP-B (not shown) and pro-SP-B reduced, n = 4 for SP-B non-reduced)	1 (25)	4 (100)	2 (50)	2 (14)	13 (93)	14 (100)	3 (21)	0 (0)	0 (0)

Absolute rate of detection, in brackets %. Western blots for SP-B and SP-C under non-reducing conditions, for pro-SP-Band pro-SP-C under reducing condition. Frequencies were compared by Fisher's exact test and corrected for the comparisons made within each group of molecular weights. No significant differences were detected.

The major physiologic form of pro-SP-B with a molecular weight of 24–25 kDa was found in the lavages of all 21 patients with CF, 14 of 15 children with chronic bronchitis, 12 of 14 patients with pneumonia and 13 of 14 children and young adults from the control group (overall in 94%) (Fig. 1A).

**Figure 2:**
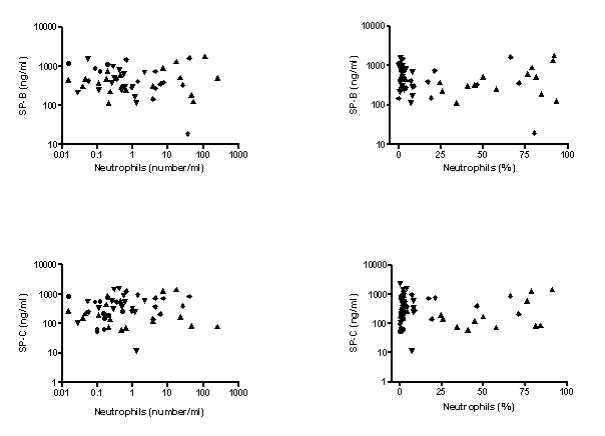
**Association of SP-B and SP-C determined under non-reducing conditions with the absolute number of neutrophils (left figures) and the percentage of neutrophil granulocytes in BALF (right figures).** ( bronchitis,  pneumonia,  CF,  controls).

**Figure 3:**
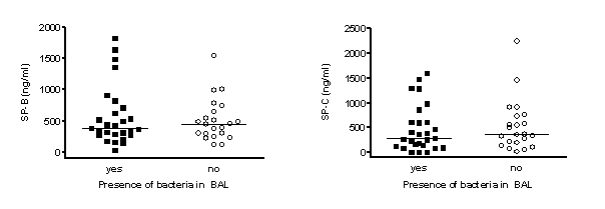
Comparison of (a) SP-B and (b) SP-C determined under non-reducing conditions between lavage samples positive or negative for the culture of bacteria.

The level of SP-B was not associated with the degree of neutrophilic inflammation or the presence or absence of bacteria in BALF (Fig. 2, Fig. 3).

Quantitatively there were no significant differences between the four groups of patients investigated (Fig. 3). Because of shortage of BAL fluid in the healthy controls, only 4 patients were analysed under non-reducing conditions, and the results under reducing conditions were given in addition.

### Surfactant protein C

61 of 64 patients had SP-C in their lavages (Table 2). In the samples CF 20-1 and CF 21-1 of the patients CF 20 and CF 21 (CF-group) and the patient P 14 (pneumonia-group) SP-C could not be detected. With 92% (CF 20-1), 93.1% (CF 21-1) and 80% (P 14) neutrophil granulocytes in BALF, these patients had the highest levels of pulmonary inflammation of their groups at the time of the lavage. In contrast, in the BALF-samples CF 20-2 and CF 21-2, which were taken from the patients CF 20 and CF 21 at a time were they had a lower level of pulmonary inflammation (57% and 43% neutrophiles), SP-C was regularly detected (Fig. 1B).

Pro-forms of SP-C were not found in any of the analysed samples. Only the Western blots of the samples CF 20-1 and CF 21-1 showed a reaction with the pro-SP-C-antibody at a molecular weight between 3 and 4 kDa; this was not observed in the blots of the samples CF 20-2 and CF 21-2, taken later (Fig. 1C). It is likely that this represents proteolytically degraded pro-SP-C, consisting of pro-SP-C peptide reactive with pro-SP-C antibody.

**Figure 4:**
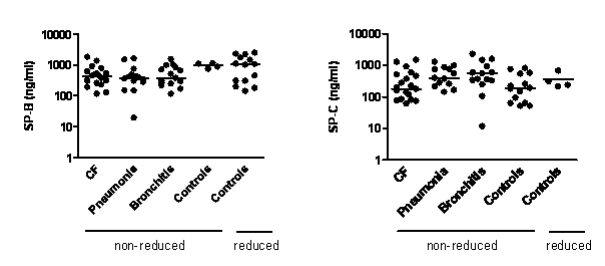
**Quantitative comparison of SP-B and SP-C, expressed per ml BAL fluid and obtained from analysis of Western blots of SP-B and SP-C under non-reducing and in the case of controls also und reducing conditions. **By Kruskal Wallis Anova no significant differences were found between the four groups of subjects. Horizontal bar indicates median.

Between the four evaluated groups no significant differences were found in the quantity of SP-B and SP-C (Fig. 4). When comparing the quantitative results of SP-C from samples analysed under reducing with those analysed under non-reducing conditions, slightly higher amounts of protein were detected under non-reducing conditions (not shown). This could either indicate that the used antibodies were less sensitive for the detection of SP-C in reduced forms, or that protein was destroyed during the reducing process. Similarly as described before for SP-B, because of shortage of BAL fluid in the healthy controls, only 4 patients were analysed under reducing conditions (Fig. 4).

Similarly as for SP-B, SP-C concentration in BALF was not related to the degree of neutrophilic inflammation or the presence or absence of bacteria in BALF (Fig. 2, Fig. 3). For both SP-B and SP-C, no age-dependencies were observed.

In addition to expressing the quantitative results per ml of lavage fluid, we also expressed all results per mg of lavage protein. Equal results to those mentioned above were obtained.

## Discussion

Here we present the results of the first systematic comparison of the molecular organisation and concentration of SP-B or SP-C and their pro-forms in BALF in children with cystic fibrosis, chronic bronchitis and pneumonia in comparison to controls. SP-B was detected regularly at molecular weights of 8 kDa in monomeric respectively 16–18 kDa in dimeric form, additional partly at approximately 30 kDa under non reducing conditions, suggesting the presence of oligomers in smaller amounts. SP-C was mainly detected at molecular weights of approximately 4.2 kDa. Some western blots also showed a band at approximately 7 kDa, which could either be caused by SP-C-dimers or covalent bonds between SP-C and other thioles [27,28]. Pro-SP-B was found in 60 of 64 samples and was therefore commonly abundant in all groups, which corresponds to previous results [10]. Pro-SP-C was not detected apart from two lavages of CF patients with very high neutrophil count. No major qualitative or quantitative differences were found between the four groups of children evaluated, suggesting that both chronic (cystic fibrosis, chronic bronchitis) and acute (pneumonia) neutrophilic inflammation is not associated with pronounced regulatory changes in these hydrophobic surfactant proteins.

This result is surprising, especially for SP-C, as recently in various animal models of lung injury the regulation of SP-C expression by inflammation has been highlighted [1]. Although we found no evidence for down-regulation of SP-C expression by pulmonary infection and inflammation, SP-C was not detected in the samples of three patients with very high percentages of neutrophile granulocytes in their lavages. However lower molecular weight pro-SP-C reactive fragments were detected, suggesting proteolysis. This was confirmed by re-lavages at later time points, where less neutrophilic inflammation and normal SP-C were found. As for SP-B, the quantitative analysis of SP-C under non reducing conditions did not show a correlation between the level of neutrophilic inflammation and the amounts of SP-C in the analysed lavage samples. These data suggest loss of SP-C by proteolysis with severe inflammation, but not down-regulation from inflammation.

In adults, SP-C content in large aggregate surfactant remained unchanged in pneumocystis pneumonia, but decreased significantly in ARDS and other pneumonia [16]. This may have also been due to proteolytic cleavage; however proteolytic activity and cleavage products were not investigated in that study. Previous results in children with malignancies and immunosuppression during fever and pulmonary infiltrates [24], and subjects with cystic fibrosis [13,14] indicated relatively small increases of SP-C levels, contradicting the view of inflammation induced down-regulation. Thus the result of the present cross sectional study over a broad range of acute and chronic neutrophilc inflammation clearly excludes the hypothesized changes. However in all these patients, changes with time and a dependency on the state of infection or inflammation might be possible and needs to be assessed, similar as has been done in many of the animal models [1].

Another issue concerns the distribution of SP-B and SP-C in the alveolar surfactant. Due to shortage of material the lavages were not separated into large surfactant aggregates and small surfactant aggregates. However from previous measurements we know that SP-C is mainly associated with the large aggregates, whereas SP-B is also present in the small aggregates, even to a larger extent as might be anticipated (Griese et al, unpublished). We did also not perform surface tension measurements of the surfactant in the lavages. From previous studies it is known that the impairment in biophysical activity associated with airway inflammation was not associated with the concentrations of SP-B and SP-C [14].

## Conclusion

Apart from the loss of SP-C in the samples of three patients with very high amounts of neutrophile granulocytes in their lavages, which was probably due to proteolysis, no significant correlation could be observed between the level of neutrophilic inflammation and the amounts of SP-B and SP-C in the lavage samples. In conclusion our data do not support, if not contradict, the notion of down-regulation of SP-B and SP-C during acute and chronic bronchial infection and inflammation in the investigated human lung diseases.

## Competing interests

The author(s) declare that they have no competing interests.

## Authors' contributions

OT carried out the laboratory research and the patients characterisation for the classification of the different patient groups and contributed to the manuscript. PL coordinated the study, participated in writing of the manuscript and assisted in performing the statistical analysis. KP recruited the CF-patients and helped with study design. MW assisted in the patients' characterisation, in laboratory analysis and interpretation of results. TW assisted in the patients' characterisation. MG conceived and supervised the study as head of the lung research group, participated in its design and coordination and wrote the manuscript. All authors read and approved the final manuscript.

## Pre-publication history

The pre-publication history for this paper can be accessed here:


